# Insomnia Symptoms and Chronic Pain among Patients Participating in a Pain Rehabilitation Program—A Registry Study

**DOI:** 10.3390/jcm10184040

**Published:** 2021-09-07

**Authors:** Josefine Lind, Paulin Andréll, Anna Grimby-Ekman

**Affiliations:** 1 Chronic Pain, School of Public Health and Community Medicine, Institute of Medicine, Sahlgrenska Academy, Gothenburg University, 405 30 Gothenburg, Sweden; josefine.lind@gmail.com; 2 Department of Anaesthesiology and Intensive Care Medicine/Pain Centre, Sahlgrenska University Hospital, Region Västra Götaland, 416 50 Gothenburg, Sweden; paulin.andrell@vgregion.se; 3 Department of Anaesthesiology and Intensive Care Medicine, Institute of Clinical Sciences at the Sahlgrenska Academy, Gothenburg University, 413 45 Gothenburg, Sweden; 4 Biostatistics, School of Public Health and Community Medicine, Institute of Medicine, Sahlgrenska Academy, Gothenburg University, 405 30 Gothenburg, Sweden

**Keywords:** chronic pain, insomnia, multimodal pain rehabilitation, interdisciplinary treatment, rehabilitation, biopsychosocial, registry study

## Abstract

Insomnia and chronic pain are prevalent health complaints. Previous research has shown that they are closely associated, but their interaction and causality are not completely understood. Further research is needed to uncover the extent to which a treatment strategy focusing on one of the conditions affects the other. This study aimed to map the prevalence of insomnia symptoms among patients in interdisciplinary pain rehabilitation program (IPRP) and investigate associations between the degree of insomnia at baseline and the treatment outcome regarding pain intensity, physical function, social function, mental well-being, anxiety, and depression. Of the 8515 patients with chronic pain, aged 15–81 who were registered in the Swedish Quality Registry for Pain Rehabilitation during 2016–2019 and participated in IPRP, 7261 had follow-up data after treatment. Logistic regression analysis was used to investigate associations. The prevalence of clinical insomnia, according to Insomnia Severity Index (ISI), among chronic pain patients in IPRP was 66%, and insomnia symptoms were associated with both country of birth and educational level. After IPRP, the prevalence of clinical insomnia decreased to 47%. There were statistically significant associations between the degree of insomnia symptoms before IPRP and physical function (*p* < 0.001), social function (*p* = 0.004) and mental well-being (*p* < 0.001). A higher degree of insomnia symptoms at baseline was associated with improvement after IPRP. In conclusion, IPRP seem to have beneficial effects on insomnia symptoms in chronic pain patients. Nevertheless, almost half of the patients still suffer from clinical insomnia after IPRP. The possible effect of systematic screening and treatment of insomnia for improving the effect of IPRP on pain is an important area for future research.

## 1. Background

Chronic pain is a widespread health problem throughout the world, and is one of the most common causes of sick leave in Sweden [1]. Globally, about 20 percent of the adult population suffers from chronic pain [2]. Chronic pain is defined as pain that lasts or recurs for more than three months and differs from acute pain, which is caused by tissue damage or imminent tissue damage (nociceptive pain) [3]. The pain system has a functional, protective role in acute pain conditions; the acute pain is a warning signal to change our behavior in order to reduce the risk of injury in the long term. The link between pain and learning and memory is therefore important for our survival and is beneficial in acute pain, but can have negative consequences in chronic pain. In contrast to acute pain, chronic pain usually has no value as a “warning signal” of ongoing tissue damage. In addition, pain mechanisms other than nociception, i.e., nociplastic and neuropathic pain mechanisms, are usually present in chronic pain conditions [4].

Chronic pain often affects mental health negatively, thus many patients also suffer from psychiatric co-morbidity in terms of anxiety, depression, stress and sleep disorders. The relationship between pain and mental health appears to be bidirectional, via the pain modulatory systems in the central nervous system (i.e., descending pain inhibitory systems and descending pain facilitating systems) psychological factors such as mood, anxiety level, memories, attention and distraction, stress, fatigue, and expectations can either increase or decrease the pain experience [5].

The bidirectional relationship between sleep and pain is of special interest. Poor sleep is common in chronic pain patients and recent data identifies sleep problems as key factors in the patients with severe pain presentations [6,7]. In a large, recent cross sectional study investigating insomnia in Norwegian adults, the prevalence of insomnia in the complete cohort was 14% [8]. In patients with musculoskeletal pain, the odds ratio (OR) for insomnia, in a fully adjusted model, was 1.7 as compared to the remaining cohort. In fibromyalgia, the OR was 2.7. In a registry study of patients undergoing pain rehabilitation, 41% of the patients fulfilled the criteria for clinical insomnia and 24% suffered from severe clinical insomnia according to the Insomnia Severity Index questionnaire [9].

Studies suggests that disturbed sleep is an important modulator and cause of perceived pain [6,10]. Examples include longitudinal studies showing that sleep impairments reliably predict new incidents and aggravation of chronic pain [11,12,13]. Other studies suggest that impaired sleep is a stronger, more reliable predictor of pain than pain is of sleep impairment [14]. On the contrary, good quality of sleep is associated with improvement in chronic widespread pain states [15].

Thus, there seem to be a bilateral connection between pain and sleep disturbance, even though the exact mechanism of action still needs to be eluded [6].

Insomnia is a symptom comprising decreased quality or quantity of sleep, despite adequate attempts to sleep or maintain sleep. It affects about 10% of the global population [16]. Insomnia can either be categorized into acute or chronic, or according to which part of the sleep cycle is most affected: falling asleep, maintaining sleep, or waking up early. In addition, insomnia may be categorized by primary or secondary cause, where primary insomnia means that there is no psychiatric underlying condition or other medical condition that can explain the insomnia [17]. To date, behavioral treatments is recommended as first line of treatment for insomnia whenever possible, whereas sleep medications should be limited to the lowest necessary dose and shortest necessary duration [16].

The Insomnia Severity Index (ISI) is a validated self-assessment tool for insomnia symptoms. It consists of seven questions giving a total score of 0–28 points that can be categorized as follows: no insomnia (0–7 points), sub-threshold insomnia (8–14 points), moderate insomnia (15–21 points), and severe insomnia (21–28 points) [18]. An ISI score of more than 10 points suggests an increased risk of developing insomnia, and can be a useful indicator to start treatment early and thus reduce the risk of developing other conditions associated with insomnia, such as chronic pain [19]. Previous studies on chronic pain and insomnia have shown that insomnia is an important risk factor for developing chronic pain [9]. It has also been shown that even a milder degree of insomnia has a negative effect on the experience of pain [10]. Previous investigations of the link between insomnia and specific pain characteristics have shown, among other things, that a large anatomical prevalence of pain is a factor with a strong link to insomnia [20].

Chronic pain is a complex medical condition in which many factors interact and affect the quality of life, such as high pain intensity, decreased mental well-being, reduced ability to work, and insomnia [21]. Patients with chronic pain can be treated via multimodal pain clinics, which means that their pain problems are treated at the same time instead of with isolated individual treatments. Interdisciplinary pain rehabilitation program (IPRP) includes physical activity/exercise and cognitive behavioral therapy, and is coordinated by an interdisciplinary team consisting of a doctor, nurse, occupational therapist, physiotherapist, and psychologist [22,23].

The focus in this type of pain rehabilitation is not primarily to reduce the pain, but to find a sustainable approach to the pain condition where reduced pain can be a positive side effect. Pain rehabilitation focuses on the patient learning strategies to be able to manage the pain and its consequences, increasing the patient’s understanding of the condition so that the patient has better opportunities to cope with daily activities and to be able to return to work.

The rehabilitation within IPRP takes place over a period of about three months, and in addition to medical and care-related measures from doctors and nurses includes rehabilitative measures at home and at work, planning and guidance of physical activity by physiotherapists, and mapping and treatment of anxiety, depression, and sleep difficulties by a psychologist.

The aim of the present project was to study insomnia symptoms among patients with chronic pain, firstly in terms of the association between the prevalence of insomnia symptoms and sociodemographic factors or pain characteristics, and secondly in terms of the association between the degree of insomnia symptoms at baseline and the treatment result regarding pain intensity, physical function, social function, mental well-being, anxiety, and depression.

## 2. Materials and Methods

Data from the Swedish Quality Registry for Pain Rehabilitation for the years 2016–2019 were retrospectively analyzed. Data in the registry were gathered via questionnaires filled in by patients on three different occasions: before start of IPRP, immediately after IPRP, and one year after end of IPRP. In the present study, the first two time points are used, before start of IPRP and immediately after. The questionnaires contain several validated self-assessment tools such as a numeric rating scale (NRS) for pain, the ISI [18], the Hospital Anxiety and Depression Scale (HADS) [24], and the RAND-36 (a modified version of the Swedish Short-Form 36 questionnaire (SF-36)) for evaluation of health-related quality of life [25]. The HADS contains seven questions each for anxiety and depression, with three categories of total score: 0–6 implies low risk of anxiety/ depression, 7–10 implies indicating risk of anxiety/depression, and >10 implies probable risk of anxiety/depression [26]. Furthermore, the patients filled out number of painful sites at the body by marking them on a list of 36 anatomical predefined areas (18 on the left side, respectively, the right side).

**Figure 1:**
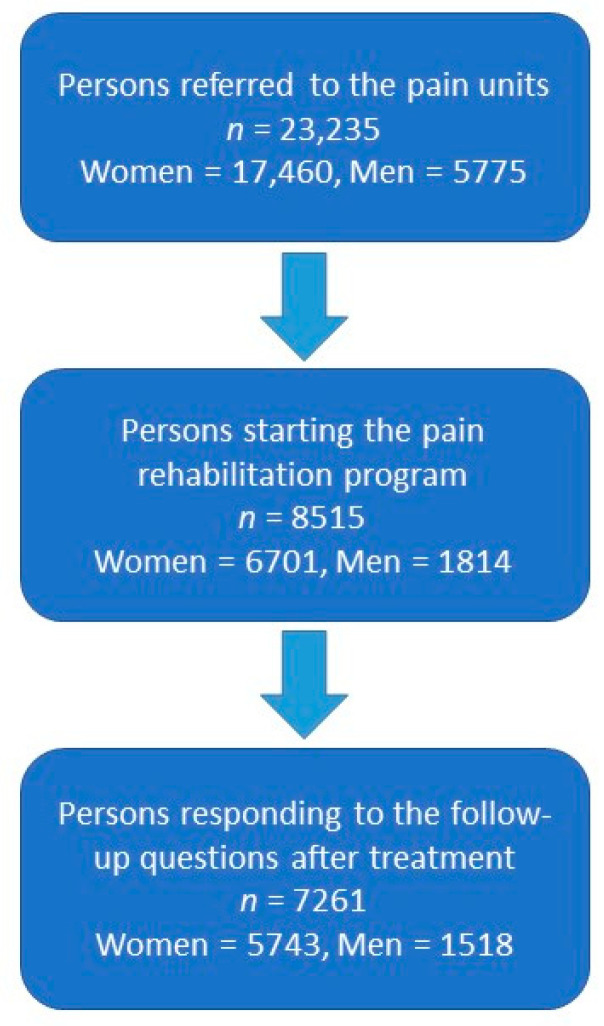
Flowchart of the study groups.

All patients included in this study were between 15–88 years old, with chronic pain treated at one of the 40 pain clinics that report to the Swedish Quality Registry for Pain Rehabilitation. For the period 2016–2019, the register contains 23,235 patients who were referred to a pain clinic, 8515 patients who were admitted to IPRP, and 7261 patients who completed the IPRP and answered the questionnaires after the IPRP. The study group at baseline for the present study consists of the patients who were admitted to IPRP, and the study group who completed IPRP consists of those who underwent IPRP and filled out the questionnaires in connection with end of IPRP. The selection process is summarized in Figure 1.

The ethics application for access to the registry in order to research multimodal pain rehabilitation was approved on 26 June 2015 (ref: 2015/108–31).

Statistical analyses were performed using the IBM SPSS^®^ Statistics software package. The ISI was used to assess sleep, with scores divided into four categories of insomnia severity (0–7, 8–14, 15–21 and 22–28), as described earlier and as used both by the developers of ISI and in research studies using the Swedish translation [9,27]. For a binary division into clinical insomnia (having clinical insomnia or not), a cutoff score of >14 points was used. This cutoff was considered the limit for clinical insomnia by the developers of the instrument [18]. Sociodemographic factors investigated were age, gender, country of birth, and education. Pain characteristic factors were pain intensity, pain frequency, pain duration, and anatomical distribution of pain. The Wilson method was used to calculate 95% confidence intervals (CIs) for the prevalence of clinical insomnia [28].

In a cross-sectional analysis of baseline data, logistic regression was used to investigate the association between the outcome of clinical insomnia and sociodemographic and pain characteristic factors. The explanatory factors were age, gender, country of birth, education, pain intensity, pain frequency, pain duration, and anatomical distribution of pain (number of pain sites). Logistic regression was also used to investigate associations between the four categories of degree of insomnia at baseline and treatment outcomes regarding pain intensity, mental well-being, physical function, social function, anxiety, and depression reported after the treatment. The treatment outcomes were defined as binary variables (improved vs. worsened), with the minimal clinically important difference (MCID) for each variable being used to calculate an improvement or deterioration. MCIDs used were 2 for pain intensity according to the NRS [29], 5 for each of the SF-36 domains [30], and 1.7 for each of anxiety and depression according to the HADS [31]. The recoded binary variables were slightly different for the variables anxiety and depression, in that the two categories “improved” and “worsened” also included “unchanged lack of anxiety/depression” and “unchanged anxiety/depression” in each category. The reason for this was that the HADS includes levels for defining no depression and no anxiety. Hence, if a patient had no change between before and after treatment and a level ≤7, they were placed in the “improved” category, while if they had no change before and after treatment and a level of >7, they were placed in the “worsened” category.

The following procedure was used for both the logistic regression analyses. No collinearity was seen between the explorative variables. This was checked with paired Spearman correlations for all variables, but also with box plots for pairs of a categorical and a continuous variable and pairs of two categorical variables. Separate logistic regression analyses were performed with one explanatory variable at a time, and with insomnia as the dependent variable. The variables that fell out with *p* < 0.2 were then put together in a multivariable model, and variables with *p* ≥ 0.2 were removed from the model one at a time according to their *p*-value.

## 3. Results

### 3.1. Baseline

The study group at baseline consisted of 8515 people, of whom 21% were men (*n* = 1814) and 79% were women (*n* = 6701). The mean age was 45 years (standard deviation [SD]: 11, range: 15–88) in the total group, 46 years (SD: 12) among the men, and 45 years (SD: 11) among the women. Further background data on the study group at baseline are given in Table 1.

In the study group at baseline, the total prevalence of clinical insomnia was 66%, the prevalence of moderate insomnia was 40%, and the prevalence of severe insomnia was 26% (Table 2). The prevalence were similar for men and women, with overlapping confidence intervals.

The logistic regression analysis with clinical insomnia as the dependent variable, using “no insomnia” as the reference category, revealed several statistically significant findings (Table 3). Most of the demographic variables and pain-related variables were statistically associated with pre-treatment clinical insomnia, with the exception of gender, where no significant associations were seen.

### 3.2. Associations of Insomnia at Baseline and Outcomes at the Follow-Up

The study group completed IPRP consisted of 7261 people, of whom 21% were men (*n* = 1518) and 79% were women (*n* = 5743). The mean age was 45 years (SD: 11, range: 15–88) in the total group, 46 years (SD: 12) among the men, and 45 years (SD: 11) among the women.

Table 4 presents descriptive statistics on the prevalence of insomnia in the study group completed IPRP. The total prevalence of clinical insomnia was 47% (95% CI: 46–48), the prevalence of moderate insomnia was 32%, and the prevalence of severe insomnia was 15% according to ISI criteria. There was no significant difference between the sexes. Compared with baseline, the total prevalence had decreased by nine percentage points from 66% to 47% (see Table 2 for prevalence of baseline insomnia). Table 5 presents descriptive statistics on anxiety, depression and the SF-36 domains in the patient group after the IPRP.

Logistic regression analysis with treatment outcome (improvement/deterioration) as a dependent variable and degree of insomnia before treatment as an independent variable showed some statistically significant results. Odds ratios for improvement in physical function, social function, and mental well-being were all higher when the degree of insomnia before treatment was higher (see Table 6 for a detailed description), while for depression and anxiety, the trend was the opposite. There was no significant difference when adjusting for confounders. No statistically significant relationship was seen between treatment outcome of pain intensity and degree of insomnia before treatment.

## 4. Discussion

The prevalence of clinical insomnia according to ISI among patients with chronic pain was high (66%) before they entered an interdisciplinary pain rehabilitation program, and although the prevalence decreased after the program, almost 50% of the patients still reported clinical insomnia. Clinical insomnia was associated with demographic factors, with depression and anxiety, and with increased severity of pain (higher number of pain sites, higher pain intensity and persistent pain). A high level of pre-treatment insomnia was associated with improvement in functional outcomes, but with non-improvement in mental health.

Our finding of a high prevalence of insomnia in people with severe chronic pain is in line with previous studies [9,32], as is our finding of a high prevalence after the IPRP [33], albeit decreased. In our study, most of the pre-treatment demographic and pain-related variables were statistically significantly associated with pre-treatment clinical insomnia, with the exception of gender, where no statistically significant association was seen. Patients at Swedish pain clinics born in non-Nordic countries had higher odds of clinical insomnia than patients born in Nordic countries, which could be of important clinical significance. Previous research on patients with chronic pain has shown associations between other sociodemographic factors and insomnia [34]. Association with country of birth and chronic pain has been seen in population studies [35]. However, the association with insomnia symptoms in our findings needs to be further explored in future clinical studies. In our results, age and educational level were statistically significantly associated with pre-treatment clinical insomnia. To our knowledge, few studies have investigated insomnia related to other pre-treatment factors, although some have examined other associations such as pain, mental health, or emotional aspects in connection with health-related quality of life [9,20].

Our results showed that a higher degree of pre-treatment insomnia was associated with higher odds of improvement in physical function, social function, and mental well-being. One possible explanation may be related to those with a higher degree of pre-treatment insomnia having a higher potential for improvement. This could be investigated in the future by analyzing whether the people who improved in these areas, and had a high degree of pre-treatment insomnia, also improved with regard to insomnia.

However, our results showed that a high degree of insomnia before treatment meant lower odds of improvement regarding depression and anxiety. Previous research has shown a strong link between anxiety and depression and insomnia [36,37]. An explanation for the findings in our analysis may be precisely this connection. It would be reasonable to assume that people with a higher degree of insomnia before treatment also have a higher degree of anxiety and depression [9,38]. The approximate three months of treatment time within IPRP is also a relatively short time for changes to occur in conditions such as anxiety and depression. There was no statistically significant relationship between the degree of pre-treatment insomnia and treatment outcome regarding pain intensity. Further statistical analysis to draw additional conclusions about chronic pain and insomnia could compare improvement within the selected variables with improvement of insomnia, in order to account for possible connections between improvement of insomnia and improvement within factors concerning chronic pain.

One of the strengths of this project was the relatively large number of people whose data could be analyzed. The fact that the register from which the data came has good coverage of pain rehabilitation and that it is based on validated self-assessment tools such as NRS, ISI, HADS, and RAND-36 strengthens the validity of our results. Conversely, although the questionnaires used to gather data were based on well-validated self-report instruments, the use of questionnaires and self-reported conditions could be considered a weakness. Since the Swedish Quality Registry for Pain Rehabilitation only includes patient-reported outcome measures, there is no information in the registry regarding diagnoses or treatment. Hence, the impact on insomnia of specific pain diagnoses, co-morbidity and pharmacological therapy cannot be assessed in this study. Another limitation of the study is that only descriptive statistics were calculated regarding differences between men and women. However, gender was adjusted for in the regression analysis.

## 5. Clinical Implications and Future Research

This study shows that the high prevalence of clinical insomnia according to ISI in this patient group persists after IPRP. Nevertheless, both pain intensity and the prevalence of clinical insomnia were lower in the group who had completed IPRP. The findings regarding the connections between insomnia and the sociodemographic and pain characteristics, as well as the connection between the different treatment outcomes and degree of insomnia before treatment, require further research in order to be able to draw additional conclusions. However, the results indicate that chronic pain patients in IPRP with severe pain presentation in terms of high-intensity, persistent and wide-spread pain seem to be a high-risk group with regard to prevalence of clinical insomnia according to the ISI.

The results also indicate that chronic pain patients with higher degree of insomnia symptoms seem to improve more with regard to physical and social function as well as to mental well-being. Giving patients this information regarding possible treatment effects before start of an IPRP might further motivate the patients to participate in the IPRP. However, further studies are needed in order to confirm the clinical relevance of these findings as well as effects of insomnia targeted treatment in chronic pain patients undergoing IPRP. These findings raise the question of whether screening and treatment of insomnia could further improve the total effect of IPRP.

## Figures and Tables

**Table 1:** Descriptive statistics on sociodemographic factors and pain characteristics in the study group at baseline (*n* = 8515).

Variables		Women		Men		Total	
		*n*	%	*n*	%	*n*	%
Education	University	2333	40	400	26	704	37
	High school	2396	41	807	52	3203	44
	Elementary school	477	8	227	15	2733	10
	Other	604	10	106	7	710	10
country of birth	Sweden	5315	81	1420	80	6735	81
	Other Nordic	158	2	38	2	196	2
	Other European	318	5	81	5	399	5
	Other	800	12	237	13	1037	12
Pain frequency	Persistent	4939	79	1351	80	6290	79
	Periodic	1337	21	344	20	1681	21
		**Median**	**(q1, q3)**	**Median**	**(q1, q3)**	**Median**	**(q1, q3)**
Pain intensity, NRS (0–10)		7	(6, 8)	7	(5, 8)	7	(6, 8)
					
Pain duration (months)		63	(26, 165)	58	(22, 141)	62	(25, 161)
Number of pain sites (0–36)		15	(9, 22)	10	(6, 16)	14	(8, 21)
					

**Table 2:** Prevalence of insomnia in the study group at baseline (*n* = 8515), classified into four categories according to the Insomnia Severity Index (ISI). Confidence intervals (CI) were calculated with the Wilson method.

	Women			Men			Total		
ISI	n	%	95% CI	n	%	95% CI	n	%	95% CI
Severe insomnia (ISI 22–28)	1589	26	24.87; 27.07	440	26	24.14; 28.25	2029	26	25.04; 26.99
Moderate insomnia (ISI 15–21)	2488	41	39.42; 41.88	648	39	36.27; 40.92	3136	40	39.11; 41.29
Sub-threshold insomnia (ISI 8–14)	1427	23	22.27; 24.39	401	24	21.89; 25.97	1828	23	22.50; 24.38
No insomnia (ISI 0–7)	618	10	9.36; 10.87	191	11	9.94; 12.98	809	10	9.71; 11.07
Clinical insomnia (ISI > 14)	4077	67	65.40; 67.77	1088	65	62.45; 67.01	5165	66	65.14; 67.24

**Table 3:** Logistic regression analysis of the study group at baseline (*n* = 8515) with clinical insomnia as dependent binary variable (clinical insomnia: ISI >14). Sociodemographic variables and pain characteristic factors were used as independent variables after checking for possible collinearity. OR = odds ratio, 95% CI = 95% confidence interval, NRS = numeric rating scale.

Variables		*n*	OR	95% CI	*p*
Age (one year increasing aging)		6454	1.01	1.00–1.01	0.024
Gender (ref: male)		5095	1.05	0.92–1.21	0.443
Country of birth (ref: Sweden, *n* = 5854)	Nordic countries	88	1.06	0.67–1.70	<0.001
	Europe	123	2.07	1.33–3.33	
	Other	389	1.91	1.48–2.51	
Level of education (ref: elementary school, *n* = 623)	High school	2870	0.88	0.72–1.07	0.028
	University	2330	0.81	0.66–0.99	
	Other	631	1.05	0.82–1.35	
Pain frequency (ref: periodically recurring, *n* = 1362)	Persistent	5092	1.17	1.02–1.34	0.026
Pain intensity (NRS 0–10)		6454	1.25	1.21–1.30	<0.001
Number of pain sites (0–36 places)		6454	1.03	1.02–1.03	<0.001

**Table 4:** Descriptive statistics on insomnia in the study group completed IPRP, classified into four categories according to the Insomnia Severity Index (ISI). Confidence intervals (CIs) were calculated with the Wilson method.

	Women			Men			Total		
	n	%	95% CI	n	%	95% CI	n	%	95% CI
Severe insomnia (ISI 22–28)	759	14	13.2–15.1	245	17	15.4–19.3	1004	15	14.0–15.7
Moderate insomnia (ISI 15–21)	1751	33	31.4–33.9	449	32	29.2–34.1	2200	32	31.3–33.5
Sub-threshold insomnia (ISI 8–14)	1708	32	30.6–33.1	402	28	26.0–30.7	2110	31	30.0–32.2
No insomnia (ISI 0–7)	1147	21	20.3–22.5	325	23	20.8–25.1	1472	22	20.7–22.7

**Table 5:** Descriptive statistics on anxiety, depression, RAND-36 domains and pain intensity in the patient group after the IPRP. HADS = Hospital Anxiety and Depression Scale, NRS = numeric rating scale.

		**Women**		**Men**		**Total**	
		***n***	**%**	***n***	**%**	***n***	**%**
Anxiety	Low risk of anxiety (HADS 0–7)	2728	49	736	52	3491	49
	Indicating risk of anxiety (HADS 8–10)	1278	23	341	23	1619	23
	Probable risk of anxiety (HADS > 10)	1615	29	368	25	1983	28
Depression	Low risk of depression (HADS 0–7)	3342	59	834	57	4176	59
	Indicating risk of depression (HADS 8–10)	1227	22	341	23	1568	22
	Probable risk of depression (HADS > 10)	1056	19	298	20	1354	19
Domains within SF-36 (0–100 points)		**Mean** **Median**	**SD**	**Mean** **Median**	**SD**	**Mean** **Median**	**SD**
Mental well-being		61 64	20	62 64	21	62 64	20
Social function		50 50	24	54 50	25	51 50	24
Physical function		60 60	22	63 65	24	61 65	23
		**Median**	**(q1, q3)**	**Median**	**(q1, q3)**	**Median**	**(q1, q3)**
Pain intensity (NRS 0–10)		6	(4, 7)	5	(3, 8)	6	(4, 7)

**Table 6:** Logistic regression analysis of the study group completed IPRP with treatment outcome as binary dependent variable (improvement/no improvement). Degree of insomnia at baseline was the independent variable, with different degrees of insomnia set in relation to the chance of improving the various treatment outcomes (pain intensity, physical function, etc.) after the IPRP. Odds ratios (ORs) are presented both raw and adjusted for confounders. 95% CI = 95% confidence interval. ISI = Insomnia Severity Index, with four categories: ISI 1 = no insomnia (0–7), ISI 2 = sub-threshold insomnia (8–14), ISI 3 = moderate insomnia (15–21) and ISI 4 = severe insomnia (22–28).

Unadjusted Model						Adjusted for Confounders						
Variables	*n*		OR	95% CI	*p*	Variables	*n*	Confounders		OR	95% CI	*p*
Pain intensity (ref: no improvement)	6081	ISI 4 ISI 3 ISI 2 ISI 1	0.99 1.03 0.98 1	0.81–1.19 0.86–1.23 0.81–1.19	0.901	Pain intensity (ref: no improvement)	6067	Sex Country of birth	ISI 4 ISI 3 ISI 2 ISI 1	1.00 0.97 1.01 1	0.82–1.22 0.81–1.16 0.83–1.23	0.919
Physical function (ref: no improvement)	6561	ISI 4 ISI 3 ISI 2 ISI 1	1.50 1.30 1.39 1	1.25–1.79 1.10–1.53 1.16–1.67	<0.001	Physical function (ref: no improvement)	5851	Level of education	ISI 4 ISI 3 ISI 2 ISI 1	1.51 1.30 1.41 1	1.25–1.83 1.10–1.55 1.17–1.70	<0.001
Social function (ref: no improvement)	5925	ISI 4 ISI 3 ISI 2 ISI 1	1.32 1.19 1.05	1.10–1.59 1.00–1.42 0.87–1.27	0.004	Social function (ref: no improvement)	5296	Level of education	ISI 4 ISI 3 ISI 2 ISI 1	1.36 1.22 1.09	1.12–1.66 1.01–1.46 0.90–1.32	0.006
Mental well-being (ref: no improvement)	6539	ISI 4 ISI 3 ISI 2 ISI 1	1.77 1.40 1.16	1.48–2.11 1.18–1.65 0.97–1.39	<0.001	Mental well-being (ref: no improvement)	5825	Level of education	ISI 4 ISI 3 ISI 2 ISI 1	1.84 1.41 1.17	1.52–2.22 1.19–1.67 0.97–1.14	<0.001
Anxiety (ref: no improvement)	6567	ISI 4 ISI 3 ISI 2 ISI 1	0.67 0.69 0.78	0.55–0.81 0.58–0.83 0.64–0.95	<0.001	Anxiety (ref: no improvement)	5832	Age Country of birth Level of education	ISI 4 ISI 3 ISI 2 ISI 1	0.69 0.72 0.80	0.56–0.85 0.59–0.87 0.65–0.98	0.002
Depression (ref: no improvement)	6573	ISI 4 ISI 3 ISI 2 ISI 1	0.59 0.70 0.90	0.48–0.72 0.57–0.85 0.72–1.15	<0.001	Depression (ref: no improvement)	5837	Age Sex Country of birth Level of education	ISI 4 ISI 3 ISI 2 ISI 1	0.63 0.73 0.92	0.50–0.78 0.59–0.90 0.73–1.15	<0.001

## Data Availability

The data presented in this study are available on request from the corresponding author and with an ethical approval of additional use. The data are not publicly available due to “The General Data Protection Regulation” in EU.
